# Development of the prosencephalic structures, ganglionic eminence, basal ganglia and thalamus at 11 + 3 to 13 + 6 gestational weeks on 3D transvaginal ultrasound including normative data

**DOI:** 10.1007/s00429-023-02679-y

**Published:** 2023-09-15

**Authors:** R. Altmann, T. Rechberger, C. Altmann, L. Hirtler, I. Scharnreitner, P. Stelzl, S. Enengl

**Affiliations:** 1 Department of Gynecology, Obstetrics and Gynecological Endocrinology, Johannes Kepler University Linz, Kepler University Hospital, Altenberger Strasse 69, 4040 Linz and Krankenhausstraße 26-30, 4020 Linz, Austria; 2 Department of Pediatrics and Adolescent Medicine, Johannes Kepler University Linz, Kepler University Hospital, Altenberger Strasse 69, 4040 Linz and Krankenhausstrasse 26-30, 4020 Linz, Austria; 3 Present Address: Division of Anatomy, Center for Anatomy and Cell Biology, Medical University of Vienna, Spitalgasse 23, 1090 Vienna, Austria

**Keywords:** Fetal brain, Ganglionic eminence, First trimester, Transvaginal ultrasound, 3D ultrasound, Neurosonography

## Abstract

**Objectives:**

To show the development of ganglionic eminence, basal ganglia and thalamus/hypothalamus in week 11 + 3 to 13 + 6 by transvaginal 3D ultrasound.

**Methods:**

To visualize the prosencephalic structures surrounding the 3rd ventricle, 285 three-dimensional ultrasound volume blocks from 402 fetuses examined were selected in a prospective transvaginal 3D study to compare ultrasound images of ganglionic eminence, basal ganglia, thalamus/hypothalamus with embryological sections. In addition, measurements of the described structures were made in 104 fetuses to quantify the embryological development.

**Results:**

The sonomorphologic characteristics of ganglionic eminence, basal ganglia and thalamus/hypothalamus are described in 71% of the fetuses examined. Measurements of the structures in 57% of the fetuses, show the following results: axGE ap = 0.17 + 0.112*CRL; axGE/I = 0.888 + 0.048*CRL; axGE/BG = 0.569 + 0.041*CRL; coGE/BG = 0.381 + 0.048*CRL; coTh lat = − 0.002 + 0.135*CRL; coTh/HyT = 3.68 + 0.059*CRL; co3.V lat = 0.54 + 0.008*CRL.

**Conclusion:**

Transvaginal 3D neurosonography allows visualization and measurement of normal structures in the fetal prosencephalon at 11 + 3 to 13 + 6 weeks of gestation (GW) including details of ganglionic eminence (GE), basal ganglia (BG), and thalamus/hypothalamus (Th/HyT). Further scientific work is needed before using the results to decide on pathological changes in patients.

## Introduction

Ganglion eminence is clearly presentable at 11 + 3 to 13 + 6 weeks of gestation as a dominant but transient structure of the brain development, the origin of cell migration along the cerebral wall (Smart and McSherry 1982) and into the basal ganglia. The aim of this work is the precise description of the ultrasound image of GE and the establishment of normal values opening the possibility to delineate normal development from pathological changes (disorders of migration of syndromic (Montaguti et al. 2022), genetic or infectious origin). In addition, normal reference ranges of the surrounding structures are generated enabling the differentiation of pathologies of the thalamus/hypothalamus, basal ganglia and third ventricle (Heaphy-Henault et al. 2018).

## Materials and methods

### Design and population

This is a prospective, cross-sectional, observational study including 402 healthy fetuses whose mothers underwent first-trimester sonography and nuchal translucency measurement. This study was approved by the local ethics committee, informed consent was obtained from all patients. In addition to the routine first-trimester scan, the fetal midbrain was investigated in all fetuses with a CRL (crown rump length) between 45 and 84 mm using a Voluson E10 (GE Medical Systems) equipped with the GE RIC 6–12 D vaginal probe. The fetuses included in the study had a normal scan at 20–24 weeks or were considered healthy on clinical neonatal examination after birth. Volume acquisition of the fetal head was performed with a maximum resolution by transvaginal ultrasound, the region of interest showing only the fetal head. For the best quality of volume blocks the scan was carried out through the upper portion of the sutura coronalis at the junction in the fonticulus anterior. In 285 fetuses (71%) the target structures were sufficiently well representable from sonomorphological determination. To generate the normal values of GE, BG, Th/HyT, and third ventricle (3.v) we used 183 consecutively acquired 3D blocks from the second half of the study selecting 104 for the analysis of the measurement parameters because of their high image quality (57% of the consecutively scanned fetuses) and correlated them to crown-rump length of the fetuses to generate standard values. The mean maternal age was 33.8 years, the mean fetal crown-rump length was 63.2 mm.

### Statistics

Intra- and interobserver variabilities of *axial: GE ap, axial: GE/I lat, axial: GE/BG lat, coronar: GE/BG cc, coronar TH lat, coronar TH/HyT cc and coronar 3.V lat* were checked by intraclass correlation coefficients type (2,1)”two-way random, single measure, absolute agreement”, and by intraclass correlation coefficients type (3,1) “two-way mixed single measures, absolute agreement”.

Correlations were reviewed for continuous variables in the case of normally distributed data (check for normal distribution by Kolmogorov–Smirnov test with Lilliefors significance correction, type I error = 10%) by Bravais-Pearson correlation coefficients, otherwise by Spearman’s rank correlation coefficients.

Furthermore, the influence of *CRL* on *GE ap [axial], GE/I lat [axial], GE/BG lat [axial], GE/BG cc [coronar], Th lat [coronar], Th/HyT cc [coronar]* and *3.V lat [coronar]* was investigated by univariate linear regression analyses.

The type I error was not adjusted for multiple testing. Therefore the results of inferential statistics are descriptive only. Statistical analysis was performed using the open-source R statistical software package, version 4.1.2 (The R Foundation for Statistical Computing, Vienna, Austria).

### Ultrasound methodology

#### Orientation

**Fig. 1:**
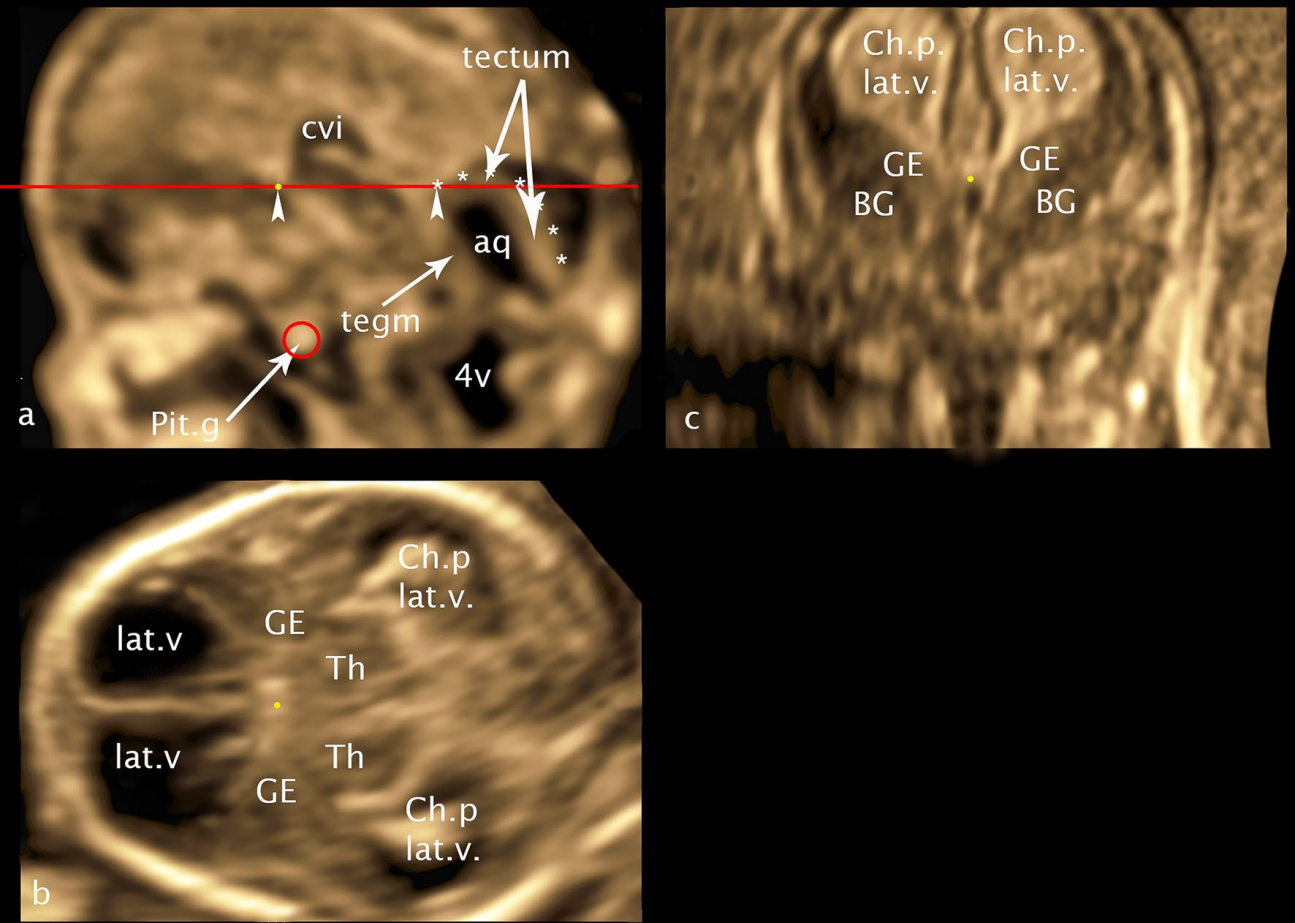
CRL 60.1 mm **a** midsagittal plane **b** axial plane** c** coronar plane: yellow point—marker dot; red circle—pituitary gland; red line—horizontal orientation line; arrowheads—orientation points for the horizontal alignment—inferior border of choroid plexus and cranial border of tectum at the entrance of the aqueduct; asterisk—cranial border of the tectum; cvi—cavum velum interpositum; aq—aqueduct; 4v—fourth ventricle; Pit.g—Pituitary gland; tegm—tegmentum; Ch.p. lat.v.—choroid plexus lateral ventricle; GE—ganglionic eminence; BG—basal ganglia: lat.v—lateral ventricle; Th—thalamus

For a better orientation in the fetal brain the midsagittal plane through the choroid plexus of the 3rd ventricle and pituitary gland was used as a reference plane in multiplanar mode additionally supplemented by a line from the inferior border of the choroid plexus of the 3.v to the cranial border of tectum serving as the horizontal orientation (Fig. 1a). Fixing this plane creates a grid of planes parallel and perpendicular to each other as a result of the multiplanar mode, which allows reproducible measurements. We will use this system of parallel and rectangular planes in our paper to further specify already defined planes on the one hand and to define a new plane on the other hand.

For the presentation and measurement of GE and BG in coronal view we used the *transcaudate plane* (Fig. 1c) (Volpe et al. 2021; Malinger et al. 2020) with choroid plexus of the third ventricle between the foramina of Monro as reference point, similarly in axial view a new *transchoroidal plane* (Fig. 1b) was defined using choroid plexus of the 3rd ventricle as craniocaudal reference point. For the measurement of the thalamus lateral and thalamus/hypothalamus (Th/HyT) in coronal view *transthalamic plane* (Volpe et al. 2021; Malinger et al. 2020) was used. For this purpose, in the grid described above, the coronal plane was moved directly behind the caudal end of the cerebral stalk.

To confirm the embryological details of the coronal section of the transcaudate plane and the axial section of the transchoroidal plane described below two new reproductions of Hochstetter's histological sections are attached (Hochstetter 1929), for the details of the transthalamic plane in coronal view, plate 11B (CRL 60 mm) of the embryological atlas of S.A. Bayer and J. Altmann is the reference (Bayer and Altman 2006).

## Results

### Sonographic findings

**Fig. 2:**
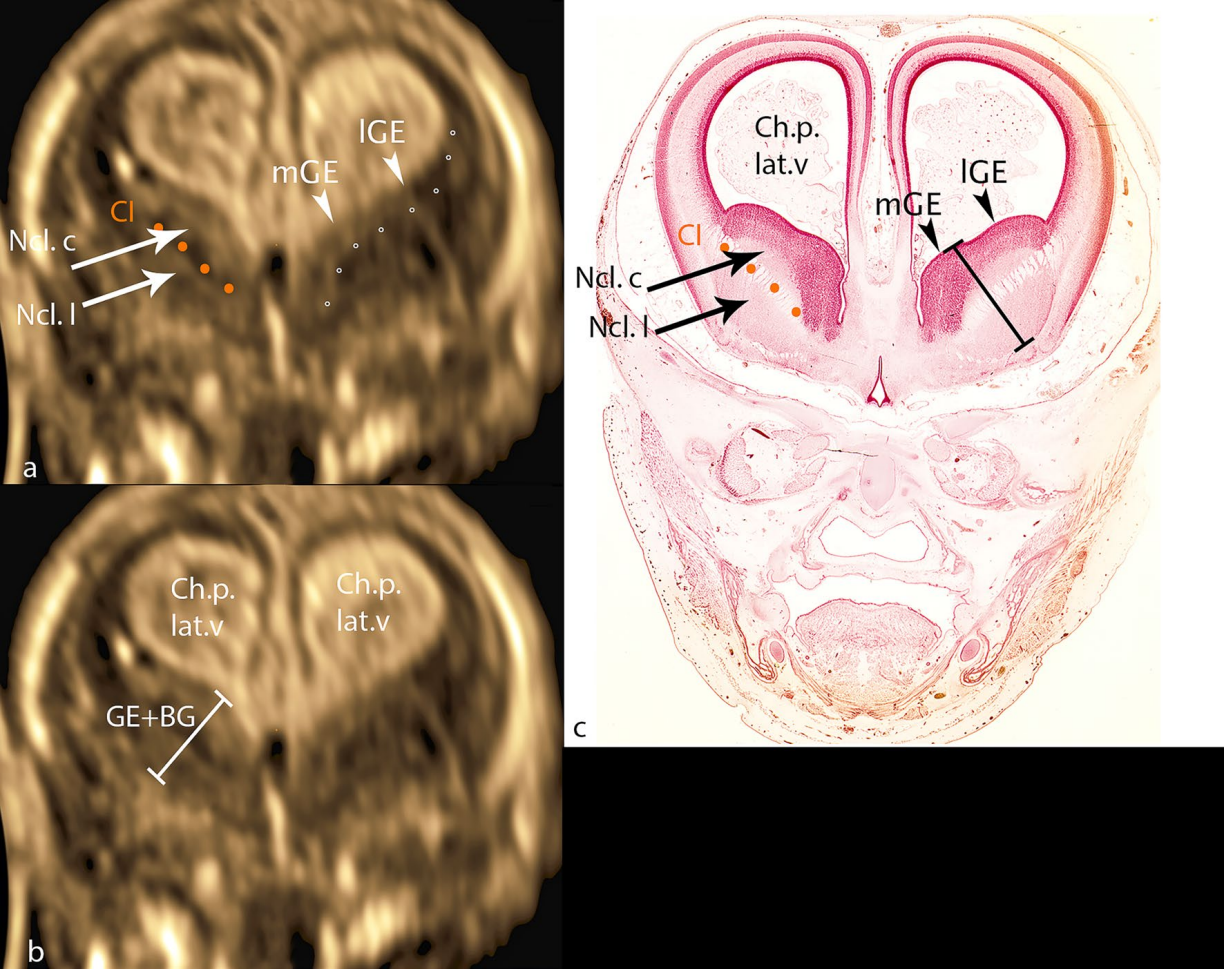
CRL 71.1 mm** a**, **b** coronar plane** c** histologic specimen (CRL 73 mm): mGE—medial ganglionic eminence; lGE—lateral ganglionic eminence; dotted line (rings)—caudal border of GE; CI—capsula interna; orange line CI—fibers of capsula interna; Ncl.c—nucleus caudatus; Ncl. l—nucleus lentiformis; GE + BG—distance ganglionic eminence + basal ganglia

In the coronary section with marker dot in the choroid plexus 3.v, the typical hyperechogenic figure of the choroid plexus in the lateral ventricle can be visualized, while in the midline, also hyperechogenic, the interhemispheric fissure can be well visualized, followed laterally by a narrow hypoechogenic zone representing the cerebral wall (Fig. 2a). Caudal to choroid plexus, frequently just below, and sometimes separated by a narrow gap of cerebral liquor, ganglionic eminences bulge prominently into the anterior horn of the lateral ventricle. The most cranially protruding point is part of medial GE, a clear separation based on different echogenicity from lateral GE not being possible. Ganglionic eminences present with median echogenicity covering the basal ganglia cap-shaped cranially, laterally narrowing towards the cerebral wall, merging into it. Medially, its boundary, corresponding to the lateral edge of the medial cerebral mantle, is difficult to delineate on ultrasound. The basal ganglia situated below GE show an elliptic figure with its caudal surface adjacent to the hypoechoic cerebral wall lying below. As a final detail, the traversing fibers of the internal capsule can be visualized, separating nucleus caudatus from nucleus lentiformis (putamen). To verify all the details described, the corresponding histological section is added using analogous annotation (Fig. 2c).

**Fig. 3:**
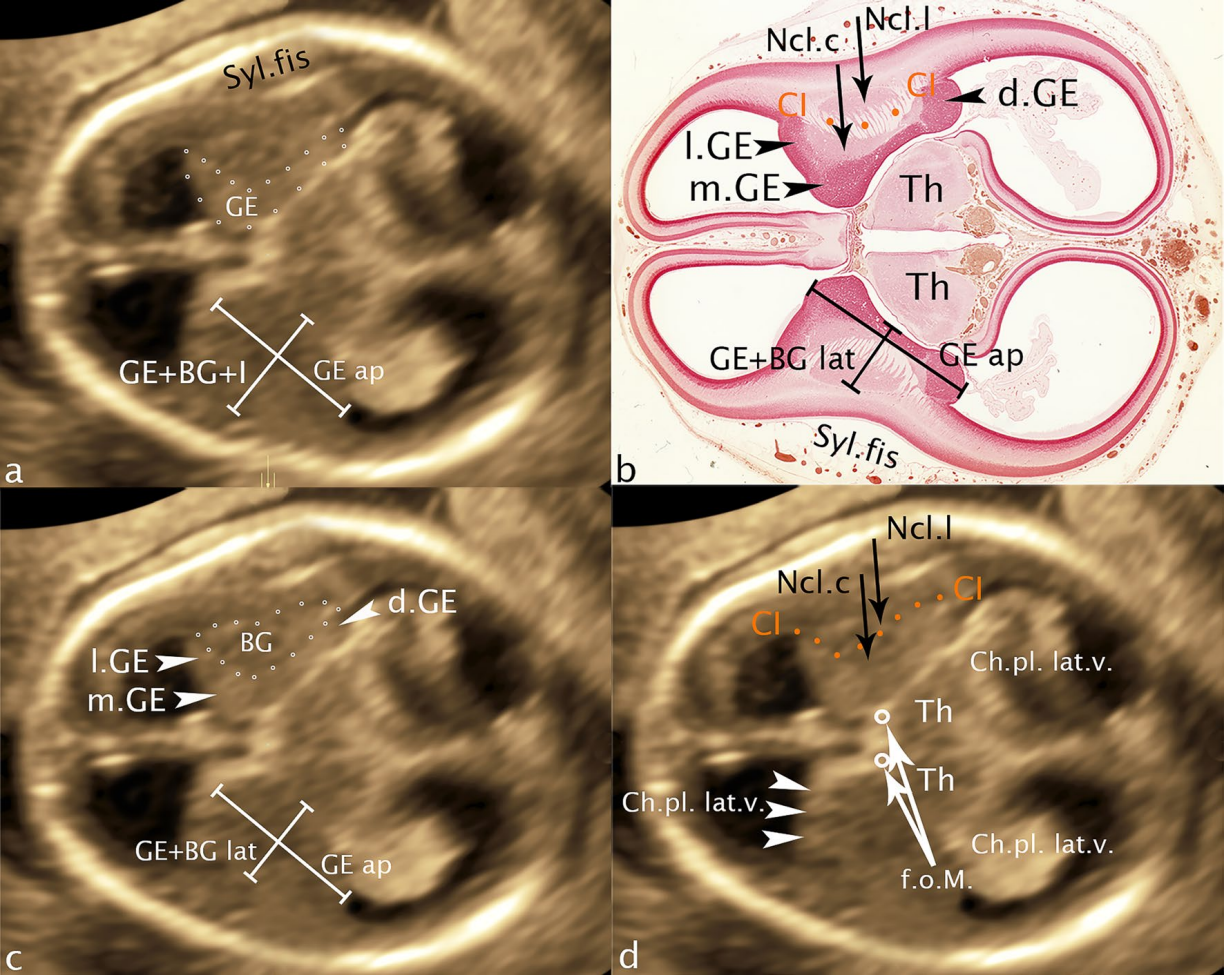
CRL 71.2 mm. **a**, **c**, **d** axial plane **b** histologic specimen (CRL 76 mm): GE—ganglionic eminence; Syl.fis—Sylivian fissure; l.GE—lateral ganglionic eminence; m.GE—medial ganglionic eminence; d.GE—dorsal ganglionic eminence; BG—basal ganglia; CI—capsula interna; Ncl.c—nucleus caudatus; Ncl.l—nucleus lentiformis; Th—thalamus; f.o.M—foramen of Monro; Ch.pl.lat.v.—choroid plexus lateral ventricle; GE + BG + I—distance ganglionic eminence/insular cortex (sylvian fissure); GE ap—distance ganglionic eminence ap; GE + BG lat—distance ganglionic eminence/basal ganglia lateral; dotted line GE—area of ganglionic eminence; dotted line (rings) BG—area of basal ganglia; orange line CI—fibers of capsula interna

In the axial plane (Fig. 3a, c, d) with marker dot analogous in choroid plexus 3.v between the foramina of Monro, the plane located just above the hemispheric peduncle, the developing neocortical wall, hypoechoic and narrow presents at the very outside in the concave curvature of the developing insula. Medially adjacent, the basal ganglia can be visualized laterally as a concave, medially as a convex structure cap-shaped again as demonstrated in the coronal plane. Analogous in this section the merging of GE into the cerebral mantle can be demonstrated frontally and temporally, the fibers of capsula interna dividing the basal ganglia into caudate and putamen (nucleus lentiformis). After the section was chosen just above the hemispheric peduncle, a narrow gap remains between the GE and the thalamus, called ganglionic narrows, filled with a small hyperechoic structure, part of the lateral ventricle correlating to the choroid plexus, its echogenicity resulting from the tangle-like arteriovenous vascular convolutes. The structures of this ultrasound section are also backed up by a new reproduction of a historical section for scientific verification (Fig. 3b). Resulting from a comparably high vascular density the subarachnoid space between calvaria and the convexity of the insula shows the same hyperechogenicity. Just caudal to the described axial plane the broad connection between the basal ganglia and the thalamus, referred to as the hemispheric peduncle is visible. On ultrasound, it presents as a massive connection between the telencephalon and the diencephalon (thalamus) well known for all the projection fibers of the developing internal capsule, including the thalamocortical fibers as the main component (Rados et al. 2006).

**Fig. 4:**
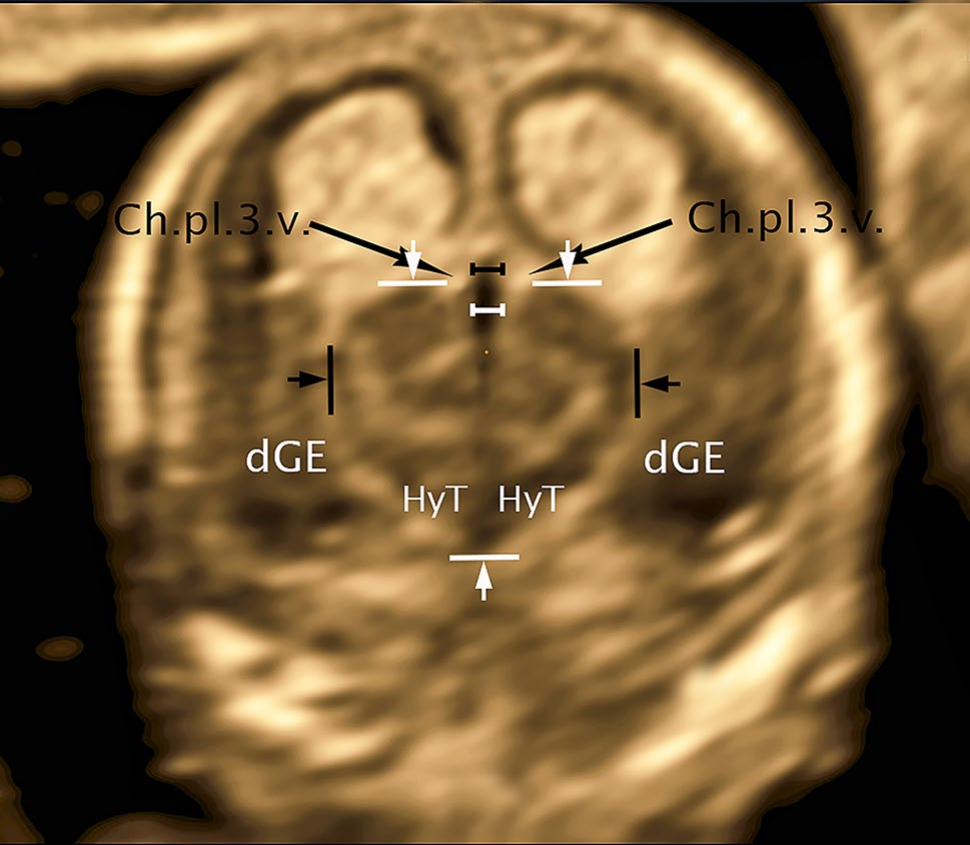
CRL 63.3 mm, coronar: Ch.pl.3.v.—choroid plexus third ventricle; dGE—dorsal ganglionic eminence; HyT—hypothalamus; distance white—distance third ventricle lateral; distance black—distance cavum velum interpositum lateral; distance arrows black—distance thalamus lat; distance arrows white—distance thalamus/hypothalamus craniocaudal

The presentation of the thalamus in the selected coronal plane dorsal to the hemispheric peduncle reveals a clear outline (Fig. 4). Cranially, the thalamus shows a strong convex bulge into the interhemispheric fissure on either side of cavum velum interpositum (CVI). The lateral border, also convex shaped, as an expression of the pronounced growth separates well from the adjacent hyperechogenic structures of Plexus choroideus of the lateral ventricle. Caudally thalamus, without a distinct limit, merges into the hypothalamus, whose caudal boundary is the hyperechogenic subarachnoid space of the base of the scull. Lateral to the narrow hyperechoic fissure, choroid plexus of the lateral ventricle, caudal ganglionic eminence presents, well demarcated from the surrounding cerebral wall. Furthermore, the full craniocaudal extent of the 3rd ventricle is well visualized, beginning with its cranial portion CVI, extending above the tela choroidea of the pl. ch.3.v into the interhemispheric fissure, showing a well-depicted lumen. Caudally the 3.v presents about the same width of the lumen becoming a fissure after a short distance reaching almost to the end of the hypothalamus. Nucleus arcuatus closes as a narrow tissue bridge, caudally adjacent to the gap of 3.v.

### Measurement

For the measurement of GE and underlying BG in an anterior posterior dimension the maximum extension of GE and BG is used in the *transchoroidal axial plane* (Fig. 3a, c). Due to the cap-shaped arrangement of GE on BG, the measurement starts anterior hypoechoic with GE analogously ending dorsal hypoechoic with GE, BG being interposed. Resulting of continuous cell migration between GE and BG, the dividing line between the two structures is not visualizable in ultrasound in some cases.

At right angles to this, a measurement between the deepest point of the insula and the medial area of GE as a total dimension (GE/Insular cortex) (Fig. 3a) and, for the first time, a measurement of GE and BG excluding the neocortical cerebral wall were performed (Fig. 3c).

In the *transcaudate coronary plane* an oblique measurement of GE and BG was made at right angles to the fibers of capsual interna, starting with the point of GE that bulges most medially into the ventricle and ending with BG at its border with the cerebral wall (Fig. 2b).

For measurement of thalamus and hypothalamus craniocaudal in the *transthalamic coronar plane*, a line was placed at the upper edge of the two cranial protrusions of thalamus right and left of the midline and defined as the cranial border, whereas the blunt caudal end of hypothalamus was defined as the caudal border, for the measurement of thalamus lateral the maximum lateral width was used (Fig. 4).

For the measurement of 3.v we also used this plane, measuring the maximum distance below the choroid plexus of the 3rd ventricle adjacent to the cranial edge of the thalamus (below CVI) (Fig. 4).

### Statistical analysis

Intraobserver variability is shown in Table 1 using Landis & Koch classification, the result showed almost perfect agreement for axGE ap, axGE/I, axGE/BG, coGE/BG, coTh lat and coTh/HyT,—co3.V showed a substantial agreement.

**Table 1:** ICC—intraclass coefficient

Parameter	ICC	95% confidence interval		*p*-Wert
		Lower limit	Upper limit	
axGE ap	0.974	0.935	0.990	< 0.001**
axGE/I	0.814	0.595	0.921	< 0.001**
axGE/BG	0.796	0.555	0.914	< 0.001**
coGE/BG	0.672	0.323	0.858	< 0.001**
coTH	0.983	0.958	0.993	< 0.001**
coTh/HyT	0.679	0.294	0.865	< 0.001**
co3v	0.632	0.284	0.834	0.001**

Interobserver variability is shown in Table 2. The interobserver variability based on 20 fetuses showed almost perfect agreement according to the parameters axGE ap, axGE/I, axGE/BG, coTh lat and coTH/HyT, the remaining parameters coGE/BG and co3.V showed a substantial agreement.

**Table 2:** ICC—interclass coefficient

Parameter	ICC	95% confidence interval		*p*-Wert
		Lower limit	Upper limit	
axGE ap	0.940	0.856	0.976	< 0.001**
axGE/I	0.801	0.564	0.916	< 0.001**
axGE/BG	0.804	0.570	0.918	< 0.001**
coGE/BG	0.795	0.553	0.914	< 0.001**
coTH	0.938	0.851	0.975	< 0.001**
coTh/HyT	0.816	0.593	0.923	< 0.001**
co3.V	0.619	0.254	0.830	0.001**

The confidence interval refers to the ICC) (Test against reliability value “0” …no agreement

**Fig. 5:**
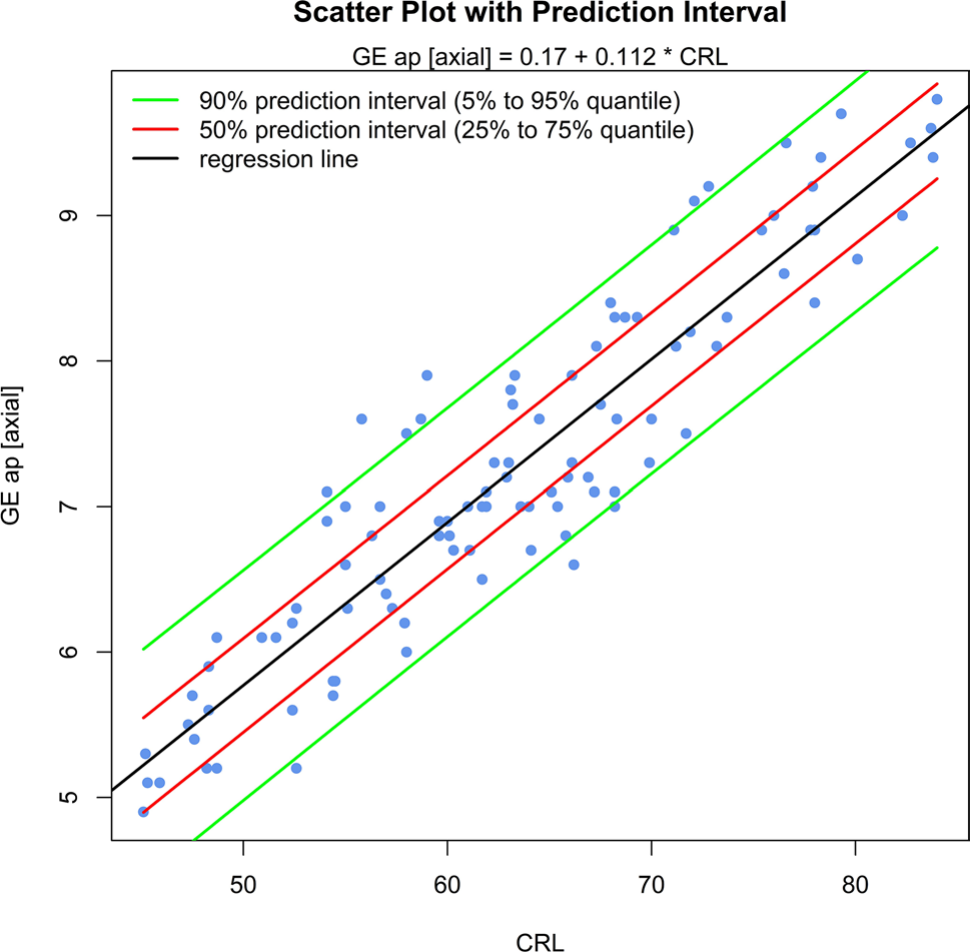
Scatter plot with prediction interval GE ap

**Fig. 6:**
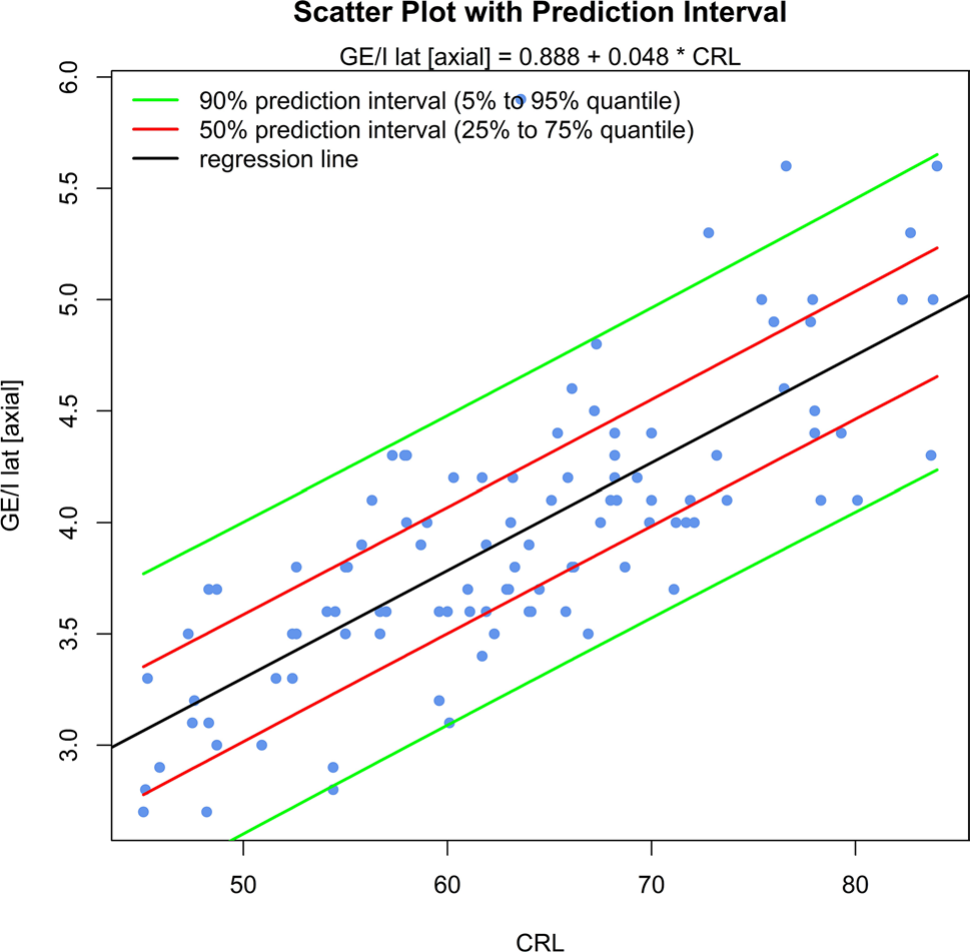
Scatter plot with prediction interval GE/I (GE + BG + I) lat

**Fig. 7:**
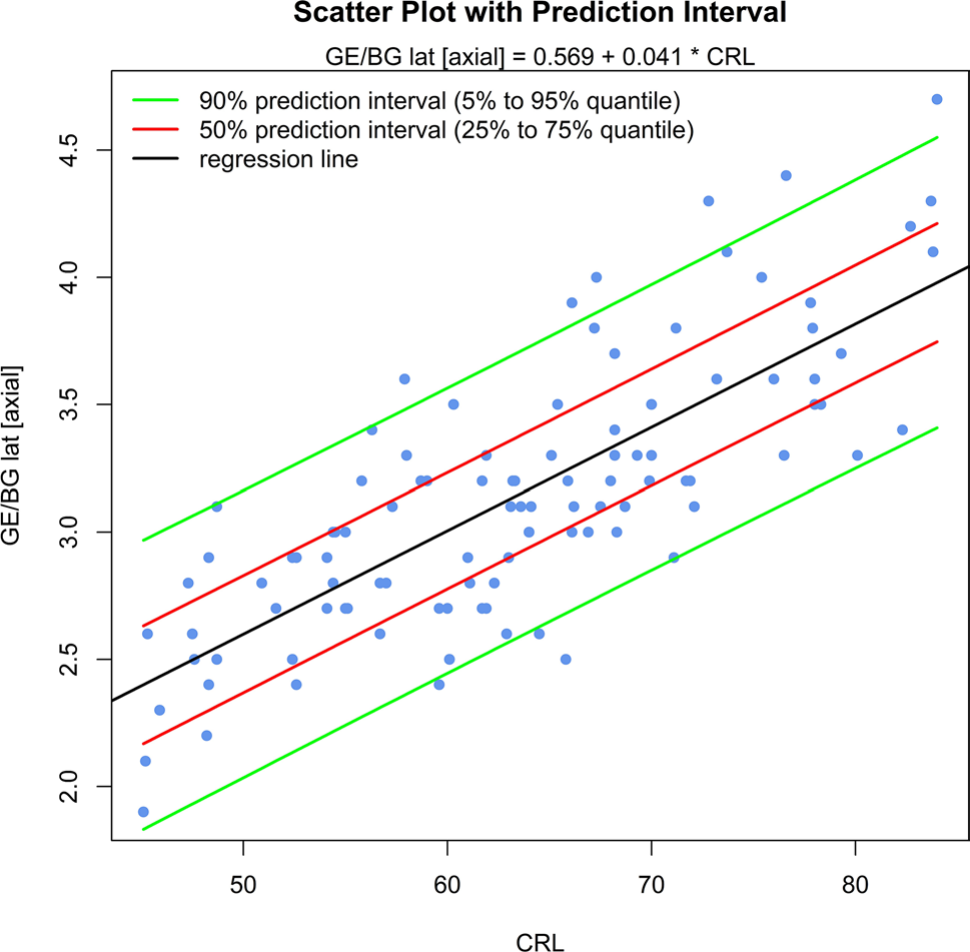
Scatter plot with prediction interval GE/BG (GE + BG) lat

**Fig. 8:**
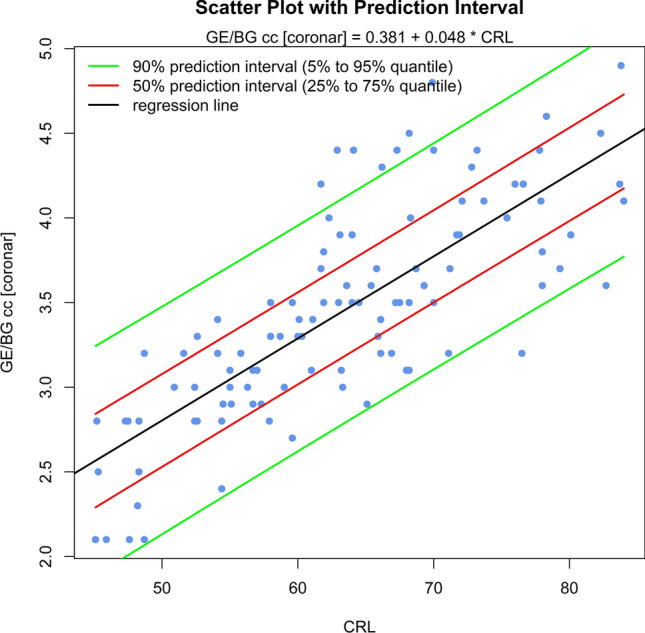
Scatter plot with prediction interval GE/BG (GE + BG) cc

**Fig. 9:**
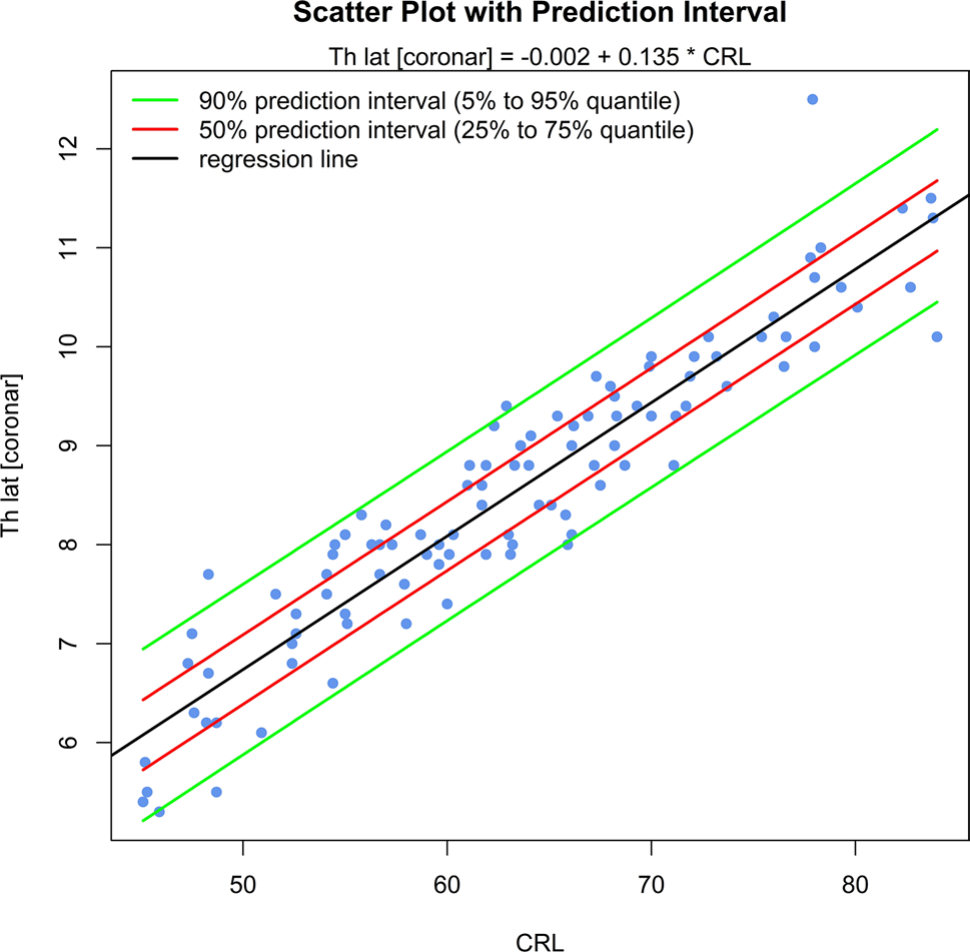
Scatter plot with prediction interval Th lat

**Fig. 10:**
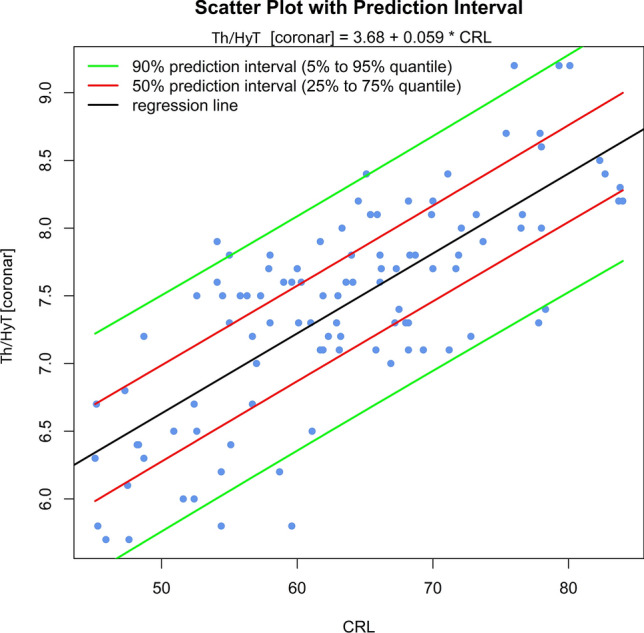
Scatter plot with prediction interval Th/HyT

**Fig. 11:**
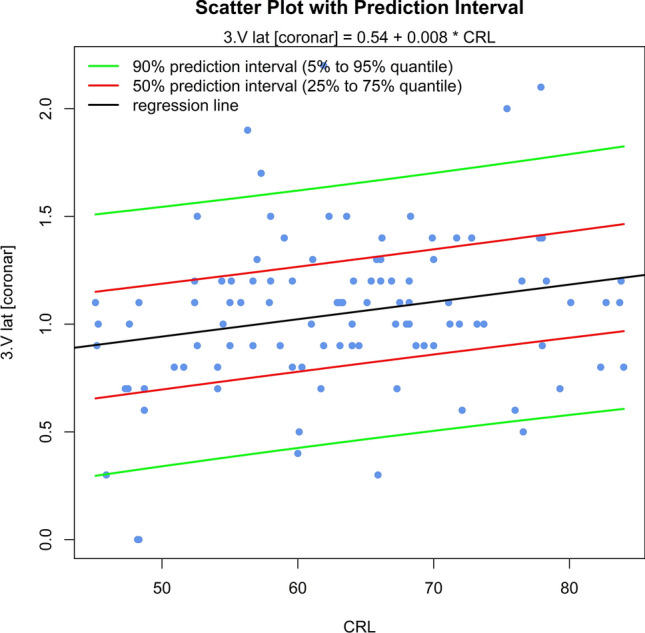
Scatter plot with prediction interval 3.V lat

Univariate regressions were created using Bravais-Pearson correlation coefficients for each of the CRL parameters as the independent variable including scatter plots with prediction intervals for better usability (all parameters except co3.V (*p* = 0.024) showing *p* < 0.001) (Figs. 5, 6, 7, 8, 9, 10, 11)

$$\begin{aligned}{\text{axGE ap }} &= \, 0.{17 } + \, 0.{112}*{\text{CRL}};\\{\text{ axGE}}/{\text{I }} &= \, 0.{888 } + \, 0.0{48}*{\text{CRL}};\\{\text{ axGE}}/{\text{BG }} &= \, 0.{569 } + \, 0.0{41}*{\text{CRL}};\\{\text{ coGE}}/{\text{BG }} &= \, 0.{381 } + \, 0.0{48}*{\text{CRL}};\\{\text{ coTh lat }} &= \, - 0.00{2 } + \, 0.{135}*{\text{CRL}};\\{\text{ coTh}}/{\text{HyT }} &= { 3}.{68 } + \, 0.0{59}*{\text{CRL}};\\{\text{ co3}}.{\text{V lat }} &= \, 0.{54 } + \, 0.00{8}*{\text{CRL}}\end{aligned}$$

### Case report

The attached case report shows a severe pathology of GE and BG (Lacalm et al. 2016; Tonni et al. 2016) highlighting the sonographic findings.

**Fig. 12:**
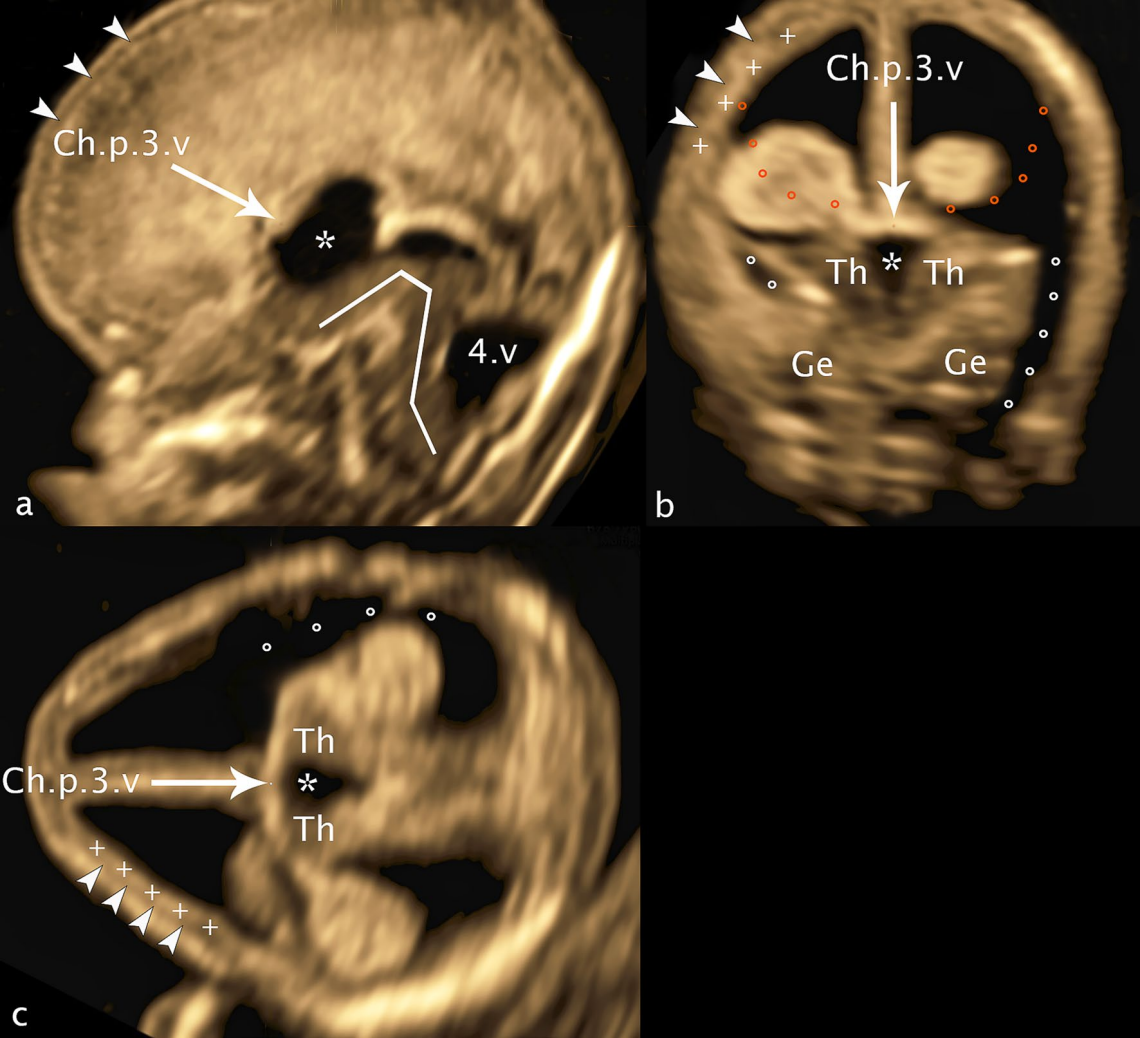
CRL 78.9 mm. **a** sagittal plane **b** coronar plane **c** axial plane: Ch.p.3.v—choroid plexus third ventricle; Th—thalamus, asterisk—third ventricle; Ge—dysplastic ganglionic eminence; dotted line (white rings)—cerebrospinal fluid surrounding dysplastic ganglionic eminence; dotted line orange—demonstrating the normally expected line of ganglionic eminence; arrowheads—reduced arachnoid space; plus—discrete band of echogenicity; white line—brainstem appearance; 4.v—enlarged fourth ventricle

**Fig. 13:**
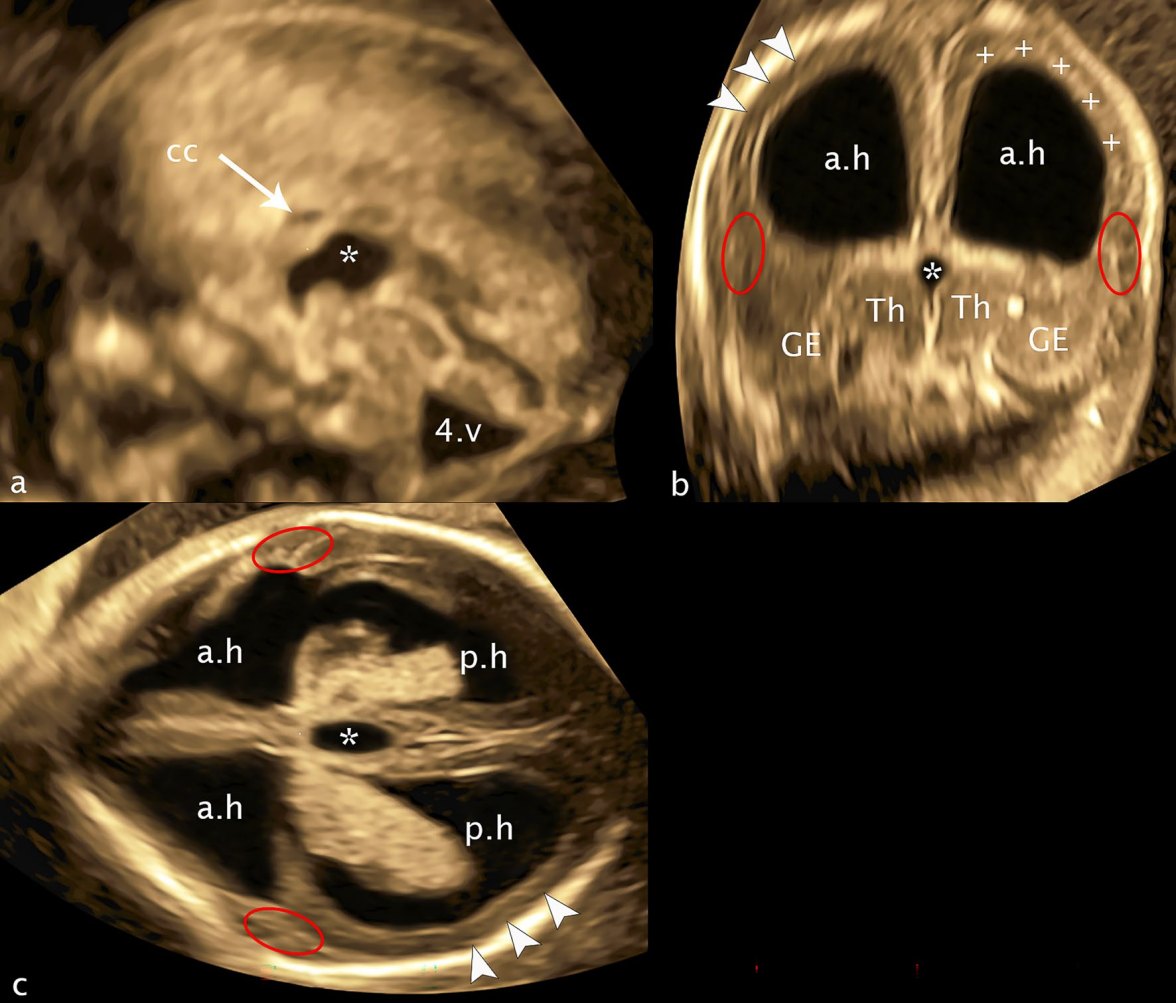
16 + 3 weeks of gestation. **a** sagittal plane **b** coronal plane **c** axial plane: cc—corpus callosum; a.h—anterior horn; p.h—posterior horn: Th—thalamus; asterisk—third ventricle; GE—ganglionic eminence; plus—band of echoghenicity; arrowheads—missed subarachnoid space; red ellipses—flat Sylvian fissure, one side bordered by a dysplastic cerebral mantle; 4.v—enlarged fourth ventricle

The intention of this case report is to show the general possibility to reveal malformations of ganglionic eminence causal for migrational disorders on ultrasound in GW 12–14, in this case accompanied by a dysplasia of the basal ganglia and the thalamus/hypothalamus at a CRL of 78 mm (Fig. 12). The sum of the brain malformations presents as a typical cobblestone lissencephaly (lissencephaly type 2), resembling the Walker-Warburg syndrome well described by Lacolm A. with a large number of brain malformations, but also differing in some details. Especially the loss of the subarachnoid space associated with the “discrete band of hyperechogenicity” can prove the diagnosis of cobblestone lissencephaly. The ultrasound changes were confirmed by targeted neurosonography at GW 16+3 after which the woman requested termination of the pregnancy (Fig. 13). Genetic testing did not reveal any changes, in particular changes corresponding to the specific phenotype (including microarray, the panel tubolinopathies, polymicrogyria, and the HPO term “basal ganglia abnormality”).

**Description**

Fig 12: In the transcaudate coronal plane, ganglionic eminence and the basal ganglia are absent on the right side, while on the left side a dysplastic structure of moderate echogenicity is visible at the level of the thalamus. In the transchoroidal axial plane the picture is mirrored analogously, the dysplastic choroid plexus is surrounded solely by cerebrospinal fluid. The slight indentation of the cerebral wall usually well presentable in this plane as an expression of the developing Sylvian fissure is also not depictable. In the transthalamic coronal plane, next to the thalamus, the upon described dysplastic structure can be identified as dysplatic basal ganglia, while on the right side between the thalamus and the cerebral mantle only cerebrospinal fluid can be visualized. Remarkable in this plane is the extended width of the 3rd ventricle (2.9 mm), while cvi is in the norm (1.3 mm). Also noteworthy is the marked increase of thalamus width (11.8 mm) with concomitant narrowing of the craniocaudal extent of thalamus/hypothalamus (6.7 mm). In addition a hypoplasia of future cavum septum pellucidum (0.9 mm ap, 1.1 mm cc), an elongation of cavum velum interpositum (6.6 mm), and an infratentorial hypoplasia of the vermis and cerebellum is part of the complex cerebral changes. A discrete band of echogenicity surrounding the surface of the brain reducing the subarachnoid space representing neuroglial overmigration into the arachnoid space is presentable in all 3D planes, in contrast the ‘Z’-shape of the brainstem as a typical sign for Walker-Warburg syndrome is missed.

Fig 13: At week 16+3, the following diagnoses could be confirmed: severe dysplasia of the ganglionic eminences and the basal ganglia and a lack of the development of fissura Sylvii, both reflecting migrational disorder, ventriculomegaly on both sides, dilated 3rd ventricle, no adhesio interthalamica, hypoplasia of the corpus callosum and an outer band of echogenicity leading to the reduced subarachnoid space complete the supratenorial alterations. Infratentorially the hypoplasia of the vermis and transcerebellar diameter complete the total number of cerebral changes.

## Discussion

The basal ganglia (basal nuclei) are symmetrical subcortical gray nuclei embryologically assigned to the prosencephalon, the most rostral of the three vesicles dividing into telencephalon and diencephalon at Carnegie stage 14 (GW 6 + 5, CRL 5–7 mm). Ganglionic eminence is one of the most important transient brain structures, cell proliferation and migration causing the development of the basal ganglia as well as the thin neocortical cerebral wall. In particular, the basal ganglia arise from two outpouchings of the ventral telencephalon, the medial ganglionic eminence (mGE), which gives rise to the globus pallidus, and the lateral ganglionic eminence (lGE), which gives rise to the caudate and putamen (Smart and McSherry 1982; Deacon et al. 1994). The ganglionic eminences are further the major source of interneurons that migrate along the pallium into the cerebral cortex (Parnavelas 2000). In 2002 caudal ganglionic eminence (cGE) was scientifically confirmed as an independent precursor region for the caudal migratory stream into the caudal cerebral cortex and hippocampus (Nery et al. 2002).

As development continues, the hemispheres grow around the diencephalon followed by a pronounced growth in size of ganglionic eminence and basal nuclei resulting in a marked widening of the hemispheric peduncle, now allowing the passage of nerve fibers that connect the hemispheres to other regions of the nervous system. The new afferent and efferent fibers developing in this embryologic period squeeze through the striated body and appear as the capsula interna. Medial to this thick medullary sheet of white matter lie the thalamus and caudate nucleus, lateral to it nucleus lentiformis consisting of the putamen and globus pallidus.

Apart from some publications on fetal cerebral midline structures GW 12–14 most researchers in recent years have focused on measurements of the lateral ventricles and the choroid plexus for the diagnosis of ventriculomegaly, genetic disorders, and spina bifida (Loureiro et al. 2012a, b; Chaoui et al. 2020).

Only one study was presented in 2017 by Dan Boitor-Borza, which confirmed the possibility to visualize GE by transvaginal 3D sonography in multiplanar and in particular in render mode in 18 fetuses at 9–13 SSW (Boitor-Borza et al. 2015). This study demonstrates the possibility to analyze GE in detail embryologically and quantified with standard values (Figs. 2a, b, 3a, c, d). Attempts to measure GE in the region extending laterally into the mantle have failed due to the impossibility of defining the lateral boundary accurately, resulting in unacceptable values for interobserver and intraobserver variability. Similarly, the isolated thickness measurement of GE was rejected because of the fuzzy transition from GE to BG.

In 1987, a study of 90 fetuses transabdominally examined in the "standard neuroanatomic plane" in GW 15–35 first established normative values for the basal ganglia measuring the distance between the edges of the thalamus and the Sylvian fissure. The measurement method (Basal Ganglia—Insula) was argued to be based on the fact that only a small portion of the insula could be allocated (Siedler and Filly 1987). The parameter GE—Insula presented and measured in this work demonstrates in contrast to the existing measurements the direct distance from GE to the outer margin of the insula and not the distance from the thalamus to the insula (Fig. 3a). Furthermore, measured values demonstrating the direct extension of GE and BG in axial (ap and lat) and coronary (cc) sections were generated (Figs. 2b, c, 3b, c). In addition, capsula interna could be visualized especially in the coronary but also in several cases in the axial section, demonstrating the subdivision of BG into its parts nucleus caudatus and lentiformis (Figs. 2a, c, 3). The clear presentation of the Sylvian fissure in the axial plane must also be mentioned in this context (Fig. 3a, b).

An early study in 1989 described the measurements of the thalamus (max ap and transverse diameter) using transvaginal evaluation (B image) in 50 fetuses in GW 12–14 (Kushnir et al. 1989). While more current scientific data reproducibly measure thalamic diameter (TD) in the transcerebellar plane at week 18–22 (Sridar et al. 2020), a volume measurement of the thalamus presented in 2011 was not shown to be useful due to its limited reproducibility (Sotiriadis et al. 2012). Our study presents the clearly defined structures of the thalamus in the coronary transthalamic plane dorsal to the hemispheric peduncle ending caudally with the hypothalamus laterally well delineated by the choroid plexus. The exact definition of the measurement plane as well as the clear separation from the surrounding structures allows unambiguous measurements of Th lat and Th/HyT cc (Fig. 4).

Using a transabdominal approach Sari in 2005 published a work describing the size and configuration of the third ventricle providing normal values for fetuses from 12 to 40 weeks of gestation, 35 fetuses in GW 12–14 (Sari et al. 2005) while Loureiro in 2012 published a work on 410 fetuses transvaginally examined presenting normative data of the roof of the third ventricle (Loureiro et al. 2012a). Implementing the knowledge of our last publication on the third ventricle and its roof developing from the cavum velum interpositum (Altmann et al. 2022) it was the intention of this work to develop a current normal value of the 3rd ventricle (Fig. 4) measured below CVI transvaginally with high resolution in addition to the already existing normal value of the roof (CVI) by Loureiro.

The latest paper to be discussed is a review by Volpe et al. (2021), summarizing all previous work, highlighting in particular the study planes that we specified in the first part of the study fixing the already known planes to clear anatomical/embryological points to ensure the reproducibility of our measurement points and to create a coordinate system that can reproducibly represent the planes in GW 12–14.

The first part of our study proves the possibility of detailed visualization of GE, BG, Th/HyT and the 3rd.V in ultrasound and their correlation to histological images. For the first time, the fibers of the internal capsule, caudate (nucleus caudatus) putamen (nucleus lentiformis), hemispheric peduncle, and caudal GE are described in detail, including a detailed description of the 3.v. in the coronal section. It can be stated that the relevant structures can be clearly visualized sonomorphologically in the described levels using targeted brain sonography.

In addition, we present scatter plots with prediction intervals for the described structures; regression analyses showed for axGE ap, axGE/I, axGE/BG, coGE/BG, coTh lat., coTh/HyT *p* < 0.001, co3.V showed *p* = 0.024.

Intraobserver variability showed almost perfect agreement for axGE ap, axGE/I, axGE/BG, coGE/BG, coTh lat, coTh/HyT—and co3.V lat showed a substantial agreement.

Interobserver variability showed almost perfect agreement according to the parameters axGE ap, axGE/I, axGE/BG lat, coTh lat and coTH/HyT lat, the remaining parameters coGE/BG and co3.V showed a substantial agreement.

As a limitation has to be mentioned that the imageability of the presented structures was sufficient in 71% of the high-resolution 3D volumes, in 57% of the fetuses the structures could be measured at least on one side. When creating the normal values, we had to face a long learning curve for identifying the limits of GE, so we now mainly recommend using the parameters GE/BG and GE/I.

This study should be seen as a basis for clarifying the diagnostic criteria of pathologic changes in the presented structures, pathologies of migration (lissencephaly, polymicrogyria, early severe CMV infections (Piccirilli et al. 2022)), dysplasia of the thalamus (mild forms of holoprosencephaly(Montaguti et al. 2022)) and dysplasia of the basal ganglia (polymicrogyria). After primary detection of abnormalities in transabdominal ultrasound experienced sonographers could in a second step by targeted transvaginal 3D ultrasound analyze the details of the prosencephalic structures to evaluate different pathologic entities.

Beyond all the data presented here for targeted transvaginal 3D ultrasound examination in GW 11 + 3 to 13 + 6, all experienced investigators should be encouraged to address the embryologic development of GE, BG Th/HyT on ultrasound. Imaging is successful in a high percentage of cases. A warning must be given against using the standard values for decisions in clinical routine; further studies, especially including pathological changes, are necessary for this purpose.

## Conclusion

We generally recommend targeted transvaginal 3 D sonography in GW 12–14 to detect early sonomorphological changes of the brain, for this work in particular of GE BG Thalamus and the 3.v. It is possible to detect severe morphological changes when knowing the normal development and the normal measurements we present, even if our measurements can only be taken as a rough guide. However it must be cautioned against making decisions based on these changes, all structures of the brain are subject to pronounced changes in development and also exhibit an increased range of variation in normality.

## Data Availability

Data and materials are available at the corresponding author.
